# Update on shoulder reconstruction in BPI

**DOI:** 10.1186/1753-6561-9-S3-A24

**Published:** 2015-05-19

**Authors:** Kazuteru Doi

**Affiliations:** 1 Department of Orthopaedic Surgery, Ogori Daiichi General Hospital, Ogori, Yamaguchi, 754 0002, Japan

## 

The shoulder joint is the foundation for excellent upper extremity function. It contributes to stability and basic movements like rotation and elevation of the arm. Shoulder fusion should not be accepted even for total palsy, and nerve repair should be recommended as long as an available donor nerve exists.

In planning the reconstructive procedure for shoulder function, accurate biomechanical analysis of individual glenohumeral (GH) and scapulothoracic (ST) joint is imperative, especially concerning serratus anterior muscle (SA) function.

Priority of nerve repair and selection of donor nerve for shoulder function are the most important key points especially in C5~7, C5~8 and total palsy depending on accompanying reconstruction of elbow and finger function including conventional nerve transfer (NT) and free muscle transfer.

Many previous articles of shoulder reconstruction with nerve repair following brachial plexus injuries were concluded from global shoulder motion that combine GH and ST motion, and emphasized only suprascapular nerve (SSN) repair. I emphasized SSN and long thoracic nerve (LTN) repair except in C56 palsy. The axillary nerve (Ax) can be ignored because simple AX palsy does not result in serious paralysis of shoulder function.

Postoperative range of shoulder motions following SSN repair, in decreasing order according to individual donor nerve was; phrenic never (PN), spinal accessory nerve (SAN), C5 root and contralateral C7 (CC7 ) root. For LTN repair; the decreasing order of post operative range of shoulder joint movements were intercostal nerve (ICN), PN, SAN, C5 root and CC7 root. PN to SSN and ICN to LTN transfer achieved the most satisfactory outcome in total palsy; however, other combinations of nerve repair and AX repair did not provide significantly better shoulder motion.

Dynamic shoulder XP is useful to differentiate between GH and ST motion for pre- and postoperative evaluation.

I will introduce our techniques of the representative nerve repairs for shoulder reconstruction.

